# Field-Based Neuromuscular and Functional Indicators Associated with Special Judo Fitness Test Performance in Young Judoka

**DOI:** 10.3390/sports14090379

**Published:** 2026-09-01

**Authors:** Artur Avelino Birk Preissler, Filipe Manuel Clemente, Marcela Zimmermann Casal, Bianca Mariante Braga, Rui Miguel Silva, Carla Gonçalves, Pedro Schons

**Affiliations:** 1 Faculdade SOGIPA, Porto Alegre 90240-485, RS, Brazilpedroschons@hotmail.com (P.S.); 2 School of Physical Education, Physiotherapy and Dance, Federal University of Rio Grande do Sul, Porto Alegre 90690-200, RS, Brazil; 3 Polytechnic University of Coimbra, 3045-093 Coimbra, Portugal; filipe.clemente5@gmail.com; 4 The Sport Physical Activity and Health Research & Innovation Center, Applied Research Institute (i2A), Polytechnic University of Coimbra, 3045-093 Coimbra, Portugal; 5 Department of Biomechanics and Sport Engineering, Gdansk University of Physical Education and Sport, 80-336 Gdańsk, Poland; 6 Escola Superior de Desporto e Lazer, Instituto Politécnico de Viana do Castelo, 4900-347 Viana do Castelo, Portugal; rui.s@ipvc.pt (R.M.S.); carlagoncalves@esdl.ipvc.pt (C.G.); 7 The Sport Physical Activity and Health Research & Innovation Center, 4900-347 Viana do Castelo, Portugal

**Keywords:** combat sports, athlete monitoring, field testing, physical performance, strength-endurance

## Abstract

Interpreting judo-specific fitness in young judoka requires practical field-based tests that reflect relevant neuromuscular and functional capacities. This cross-sectional study aimed to analyze the associations between field-based neuromuscular and functional indicators and Special Judo Fitness Test performance in young judoka. The sample comprised 36 judoka, including 27 male and 9 female athletes aged 15 to 22 years. Athletes were assessed during a beginning-of-season physical assessment session for body mass, stature, squat jump, countermovement jump, medicine ball throw without and with countermovement, judogi-grip dynamic pull-up, judogi-grip isometric pull-up, Y Balance Test score, and Special Judo Fitness Test performance. The SJFT total number of throws was treated as the primary outcome, whereas the SJFT index was treated as a secondary outcome. SJFT total was positively associated with squat jump (r = 0.670), countermovement jump (r = 0.637), judogi-grip dynamic pull-up (r = 0.514), judogi-grip isometric pull-up (r = 0.579), and Y Balance Test score (r = 0.501). Medicine ball throw performance was not significantly associated with SJFT total. An exploratory regression model including countermovement jump and judogi-grip isometric pull-up accounted for 46.7% of the variance in SJFT total performance after adjustment for model complexity (adjusted R^2^ = 0.467). These findings indicate that lower-limb power and judogi-grip pulling strength-endurance are relevant field-based indicators associated with SJFT performance in young judoka. Therefore, countermovement jump and judogi-grip isometric pull-up may support applied monitoring of physical capacities related to SJFT performance in developmental judo settings.

## 1. Introduction

Competitive judo is a high-intensity intermittent combat sport in which technical-tactical actions are executed through repeated gripping, pulling, rapid displacement, and explosive throwing efforts [1,2]. Although oxidative metabolism supplies a substantial proportion of total match energy, decisive actions also depend on rapid phosphagen and glycolytic energy provision [3,4]. Accordingly, successful preparation requires an integrated profile of lower- and upper-body strength, neuromuscular power, strength-endurance, and cardiorespiratory fitness [1,5]. In young judoka, these capacities develop alongside biological maturation, body-composition changes, and accumulated training experience, which complicates interpretation of isolated performance tests [6,7].

The Special Judo Fitness Test (SJFT) is the most extensively investigated judo-specific field test and comprises three throwing bouts of 15, 30, and 30 s separated by brief recovery intervals [4,8]. Its principal outputs are the total number of throws and an index calculated from the immediate and 1 min post-test heart rates divided by total throws [8,9]. Thus, total throws emphasizes sustained judo-specific work output, whereas the index integrates work output with cardiovascular response and early recovery [4,8]. Normative data are available for junior and senior male and female athletes, supporting classification and training monitoring across developmental and competitive levels [8,9]. However, systematic appraisal of sport-specific tests in Olympic combat sports has identified incomplete evidence for construct validity, sensitivity, feasibility, and application, indicating that SJFT results should be interpreted as one component of a broader performance profile [10].

Previous studies support a relationship between general physical fitness and SJFT performance, but the implicated determinants vary across populations and measurement approaches [11,12,13]. In experienced male athletes, upper-body aerobic power and peak anaerobic power explained only part of the variance in SJFT outcomes [11]. In athletes aged 11–16 years, upper- and lower-limb strength and power measures were associated with judo-specific tasks and provided moderate prediction of SJFT performance [12]. Other youth data indicate that maturation status, adiposity, and training experience also contribute to SJFT variability [6]. Judogi-grip dynamic and isometric chin-up tests have been standardized for cadet and junior athletes, and an 8-week strength program in cadets improved dynamic judogi chin-up performance and several SJFT outputs without improving countermovement jump height [14,15]. These findings suggest that generic and judo-specific capacities are related but not interchangeable [11,15].

Important uncertainty therefore remains regarding which low-cost field indicators provide complementary information about SJFT performance in developmental contexts [10,12]. Much of the existing evidence is based on adult men, restricted youth age ranges, laboratory ergometry, or test batteries that do not simultaneously represent lower-limb power, upper-body ballistic power, judogi-grip strength-endurance, and dynamic balance [11,12,13]. Assessing these domains concurrently enables their associations with SJFT total and SJFT index to be examined within the same athletes and analytical framework, rather than considering each physical capacity in isolation. Evidence is particularly limited on whether these field indicators relate similarly to total throws and the SJFT index, despite the different information embedded in those outcomes [4,8,13]. Clarifying these associations could support practical monitoring when coaches require repeatable assessments that complement, rather than replace, the more demanding judo-specific test [8,10].

Therefore, this study aimed to analyze the associations between field-based neuromuscular and functional indicators and SJFT performance in young judoka. Squat-jump and countermovement jump performance, medicine ball throws performed without and with countermovement, judogi-grip dynamic and isometric pull-ups, and dynamic balance were evaluated as candidate indicators. The total number of throws was specified as the primary outcome, whereas the SJFT index was treated as a secondary composite outcome. We hypothesized that lower-limb power and judogi-grip pulling strength-endurance would be positively associated with total throws and inversely associated with the SJFT index [12,13,14], while associations involving upper-body medicine ball throwing and dynamic balance were considered exploratory.

## 2. Materials and Methods

### 2.1. Participants

Participants were young judoka from the developmental and competitive training program of a Brazilian judo club. The sample comprised competitive judoka with previous experience in state, national, and international competitions. Most athletes held black belts. Among the 27 male athletes, the registered competitive weight categories represented in the sample were −50 kg (*n* = 1), −55 kg (*n* = 3), −60 kg (*n* = 4), −66 kg (*n* = 4), −73 kg (*n* = 5), −81 kg (*n* = 3), −90 kg (*n* = 3), −100 kg (*n* = 2), and +100 kg (*n* = 2). Among the nine female athletes, the registered competitive weight categories were −48 kg (*n* = 2), −52 kg (*n* = 3), −63 kg (*n* = 2), and −70 kg (*n* = 2). At the time of testing, none of the athletes were engaged in acute weight-loss procedures. Assessments were conducted during the early preparatory period. The athletes were regularly involved in the club’s training process and participated in the team’s beginning-of-season physical assessment routine. Their regular training schedule typically comprised approximately 10 sessions distributed across six days per week, with each session lasting approximately 2 h. This study was designed as an observational, cross-sectional, descriptive, and quantitative investigation conducted in an applied judo training setting. Participants were recruited using a convenience sampling approach, based on their availability to complete the assessment battery performed during the initial preparatory period for the team’s competitions. No a priori sample-size or statistical power calculation was performed because recruitment was based on the convenience sample available within the club’s routine monitoring process. Athletes were eligible for the study if they were part of the club’s developmental or competitive judo program and completed all anthropometric, neuromuscular, functional, judogi-grip strength-endurance, and Special Judo Fitness Test assessments required for the analyses. Incomplete data regarding the analyzed variables served as an exclusion criterion. Thirty-six athletes completed the entire battery of assessments and were included in the sample. The 15-to-22 age range reflected the observed ages of the athletes assessed. The sample consisted of 36 judokas: 27 male and 9 female. Detailed anthropometric, neuromuscular, functional, judogi-grip strength-endurance, and SJFT performance characteristics are presented in Table 1. This study was conducted in accordance with the principles of the Declaration of Helsinki. Written informed consent was obtained from the athletes’ legal guardians when applicable, and athletes provided assent prior to participation. Adult athletes provided their own informed consent when applicable. Ethical approval was granted by the Research Ethics Committee of the Pontifical Catholic University of Rio Grande do Sul (CEP-PUCRS) (protocol code: 7.047.156; approval date: 2 September 2024).

### 2.2. Design and Setting

Data were obtained in an applied judo training setting as part of the athletes’ beginning-of-season physical assessment routine. The assessments were conducted during the initial preparatory period for the team’s competitions, approximately one week after the athletes had returned to regular training for the season. This timing was selected because it represented the beginning of the monitoring process and allowed the coaching staff to characterize the athletes’ physical and judo-specific fitness profile before the main competitive demands of the season. The study used a cross-sectional approach, with all variables analyzed from a single assessment moment for each athlete. The assessments were performed within the athletes’ regular training environment, aiming to minimize interference with the club’s training schedule and to preserve the ecological characteristics of the evaluation process. The analytical focus was to examine whether field-based neuromuscular and functional indicators were associated with performance in the Special Judo Fitness Test. The SJFT total number of throws was treated as the primary outcome because it represents the athletes’ judo-specific throwing performance during the test. The SJFT index was treated as a secondary outcome because it integrates throwing performance and cardiovascular response.

### 2.3. Procedures

The testing battery included anthropometric assessment, dynamic balance assessment, lower-limb power tests, upper-body power tests, judogi-grip upper-body strength-endurance tests, and the Special Judo Fitness Test. At least 24 h before testing, athletes were instructed to avoid caffeine and strenuous exercise and to maintain their usual food intake and hydration. The order of the assessments followed a practical progression from simpler and less physically demanding tests to more complex and fatiguing assessments. Thus, body mass and stature were assessed first, followed by a standardized warm-up lasting approximately 10 min. The warm-up was conducted by a member of the coaching staff under the supervision of an experienced member of the research team and included upper- and lower-limb joint mobility exercises, dynamic stretching, and progressive whole-body movements commonly used in judo training routines. After the warm-up, athletes performed the Y Balance Test, squat jump, countermovement jump, medicine ball throws without and with countermovement, judogi-grip dynamic and isometric pull-up tests, and finally the Special Judo Fitness Test. For most field-based physical performance tests, two valid attempts were performed, and the best performance was used for analysis. A minimum interval of 30 s was provided between attempts, and athletes rested for approximately 3 to 5 min between tests. All assessments were conducted as part of the club’s monitoring routine and were performed in the athletes’ regular training environment. The athletes were accustomed to these assessment procedures through their previous participation in the monitoring routine. The specific procedures for each assessment are described in the following subsections. All assessments followed standardized procedures supported by the methodological references cited in their respective subsections.

#### 2.3.1. Anthropometric Assessment

Anthropometric assessment included body mass and stature. Body mass was measured using a digital scale with 0.1 kg resolution (G-TECH, Accumed Produtos Médico Hospitalares Ltda., Duque de Caxias, Brazil), with athletes barefoot and wearing light clothing. Stature was measured using a non-extensible measuring tape fixed vertically to a flat wall, with 1 mm resolution. During stature measurement, athletes remained barefoot in an upright position, with heels together and head positioned in the Frankfurt plane. Chronological age was calculated from the athletes’ date of birth and the date of assessment. Body mass, stature, and chronological age were used to characterize the sample and describe the anthropometric profile of the young judoka included in the study.

#### 2.3.2. Dynamic Balance Assessment

Dynamic balance was assessed using the Y Balance Test. The test was performed with three measuring tapes fixed to the floor in a Y-shaped configuration, allowing reach assessment in the anterior, posteromedial, and posterolateral directions. Before the test, lower-limb length was measured for the right and left limbs and used to normalize reach distances. Athletes performed the test barefoot, with their hands positioned on the waist throughout each attempt. For each trial, the athlete maintained single-leg support while reaching as far as possible with the opposite limb along the indicated direction, without losing balance or moving the support foot from the initial position. Standardized verbal instructions were provided by the evaluators before testing. Trials were considered invalid when the athlete removed their hands from their waist, displaced the support foot, used the reaching foot for support, or was unable to return to the starting position under control. Two valid attempts were performed for each direction and limb, and the best reach distance was retained for analysis. For each limb, the composite score was calculated as the sum of the best reach distances in the anterior, posteromedial, and posterolateral directions divided by three times the corresponding lower-limb length and multiplied by 100 [16,17]. The final Y Balance Test score used in the analyses was calculated as the mean of the right and left composite scores and expressed as a percentage, with higher values indicating better dynamic balance performance.

#### 2.3.3. Vertical Jump Tests

Lower-limb power was assessed using the squat jump and countermovement jump tests. Both tests were performed on a contact mat connected to a portable computer for data recording (Jump System, Cefise, Nova Odessa, Brazil). Athletes performed the tests with their hands positioned on the hips to reduce the influence of arm swing on jump performance. The squat jump (SJ) was performed first. For this test, athletes started from a static semi-squat position, with hips and knees flexed at approximately 90°, and were instructed to jump as high as possible after an audible command, without performing any countermovement. The knee angle was controlled by visual inspection from experienced evaluators. For the countermovement jump (CMJ), athletes started from an upright standing position, with hands on the hips. After the command, they performed a rapid downward movement to approximately 90° of knee flexion, immediately followed by a maximal vertical jump through concentric extension of the lower limbs [18,19]. Two valid attempts were performed for each jump condition, with a minimum interval of 30 s between attempts. The highest value obtained in each condition was retained for analysis. Jump height was determined using Jump System 1.0 software, based on flight time, and calculated using the equation h = g × t^2^/8, where h represents jump height, g represents gravitational acceleration, and t represents flight time. Results were expressed in centimeters. In the present sample, the intraclass correlation coefficient and Cronbach’s α were 0.954 and 0.976 for SJ and 0.962 and 0.981 for CMJ, respectively.

#### 2.3.4. Medicine Ball Throw

Upper-body power was assessed using medicine ball throw tests performed without countermovement and with countermovement. Athletes performed the tests from a seated position, with their backs supported against a wall and legs extended in front of the body. A 3 kg rubber medicine ball, with a circumference of 54–57 cm, was used. Before testing, standardized verbal instructions were provided by the evaluators to ensure proper body positioning and movement execution. For the medicine ball throw without countermovement (MBT-SC), athletes held the ball close to the chest and, after an audible command, threw it forward with maximal effort, without performing any preparatory movement. The ball was released approximately at eye level to standardize the projection angle and reduce excessive vertical displacement. Athletes were instructed to maintain their backs in contact with the wall throughout the movement. For the countermovement medicine ball throw (MBT-CM), athletes adopted the same initial seated position but held the ball slightly away from the chest [20,21]. After the command, they performed a rapid elbow flexion immediately before throwing the ball forward with maximal effort, maintaining a similar release height. The horizontal distance achieved was measured from the wall to the first point of ground contact of the ball. Two valid attempts were performed for each condition, with a minimum interval of 30 s between attempts. Trials were considered invalid when athletes failed to maintain the required body position or did not execute the movement correctly; in these cases, the attempt was repeated. The best result from each condition was retained for analysis and expressed in meters. In the present sample, the intraclass correlation coefficient and Cronbach’s α were 0.905 and 0.956 for MBT-SC and 0.965 and 0.982 for MBT-CM, respectively.

#### 2.3.5. Judogi-Grip Upper-Body Strength-Endurance Tests

Upper-body strength-endurance was assessed using judogi-grip dynamic and isometric pull-up tests. Both tests were performed using a judogi fixed to a horizontal bar, allowing athletes to grip the fabric in a standardized manner. Before testing, standardized verbal instructions were provided by the evaluators, and the bar height was adjusted to ensure full body suspension without ground contact. For the judogi-grip dynamic pull-up test, athletes started from a suspended position while holding the judogi, with elbows fully extended and using a self-selected grip width on the fabric. After an audible command, athletes performed repeated pulling actions through elbow flexion and extension until task failure, aiming to complete the maximum number of correct repetitions possible. A repetition was considered valid when the athlete pulled the body upward in a controlled manner and returned to the starting position with elbows extended before initiating the next repetition. The test was terminated when the athlete was no longer able to complete a valid repetition or maintain the required execution pattern. Performance was expressed as the total number of valid repetitions. For the judogi-grip isometric pull-up test, athletes maintained a suspended position while holding the judogi, with elbows flexed at approximately 90° and the chin kept above hand level [14,21]. The objective was to sustain this position for as long as possible. The test was terminated when the athlete was no longer able to maintain the required position. Performance was recorded in seconds using a digital stopwatch.

#### 2.3.6. Special Judo Fitness Test

Judo-specific fitness was assessed using the Special Judo Fitness Test. The test was performed with one evaluated athlete and two throwing partners with equivalent body mass. The two partners were positioned 6 m apart from each other, and the evaluated athlete started from the central point, approximately 3 m from each partner. Athletes were allowed to perform the throwing technique to their preferred side. After each throw, the evaluated athlete was required to return to the central point before initiating the next throwing action. Standardized verbal instructions were provided before the test, and the evaluated athlete was instructed to perform the maximum number of throws possible during each effort period. The test consisted of three effort periods, 15 s, 30 s, and 30 s, separated by 10 s intervals. One evaluator controlled the test time using a stopwatch and provided the start, interval, and restart signals using a whistle. During each effort period, the athlete repeatedly performed the throwing technique on the two partners, alternating between them and returning to the central point after each throw. Only correctly completed throws performed within each effort period were counted. The number of throws performed in each series was recorded separately as SJFT A, SJFT B, and SJFT C. The SJFT total was calculated as the sum of the throws performed across the three series. Heart rate was recorded using a chest strap heart rate monitor (Polar H7, Polar Electro Oy, Kempele, Finland). Heart rate was measured immediately after the end of the test and again one minute after the test. The SJFT index was calculated as the sum of heart rate immediately after the test and heart rate one minute after the test, divided by the total number of throws performed. Therefore, a higher SJFT total indicated better throwing performance during the test, whereas a lower SJFT index indicated better judo-specific fitness, as it represented a more favorable relationship between throwing performance and post-exercise heart rate response [14,22]. Only one valid attempt was performed, given the high-intensity and fatiguing nature of the test. The variables retained for analysis were SJFT A, SJFT B, SJFT C, SJFT total, heart rate immediately post-SJFT, heart rate one minute post-SJFT, and SJFT index.

### 2.4. Statistical Analysis

Descriptive statistics were presented as mean ± standard deviation, 95% confidence interval, minimum, and maximum values for continuous variables. The normality of continuous variables was assessed using the Shapiro–Wilk test. The SJFT total and SJFT index, as well as the main neuromuscular and functional indicators included in the primary association analyses, presented normal distributions. Therefore, associations between field-based neuromuscular/functional indicators and SJFT performance were analyzed using Pearson’s correlation coefficient. The *p*-values from the correlation analyses were adjusted using the Benjamini–Hochberg procedure within each SJFT outcome to reduce the false discovery rate due to multiple comparisons. Because HR 1 min post-SJFT did not present a normal distribution, internal associations among SJFT components were analyzed using Spearman’s rank correlation coefficient. These analyses were performed to clarify the relationship between SJFT total, HR post-SJFT, HR 1 min post-SJFT, and SJFT index. The magnitudes of correlation coefficients were interpreted using absolute values as trivial (<0.10), small (0.10–0.29), moderate (0.30–0.49), large (0.50–0.69), very large (0.70–0.89), and nearly perfect (≥0.90) [23,24]. Linear regression models were used to examine the cross-sectional associations between selected field-based indicators and SJFT performance. First, a single-predictor model including only countermovement jump was tested for SJFT total. Then, the main model included countermovement jump and judogi-grip isometric pull-up as simultaneous predictors of SJFT total, representing lower-limb power and upper-body pulling strength-endurance, respectively. A secondary model including the same predictors was tested for SJFT index. Model fit was reported using R, coefficient of determination (R^2^), adjusted R^2^, F statistic, and model *p*-value. Regression coefficients were reported using the unstandardized coefficient (B), standard error (SE), standardized coefficient (β), 95% confidence interval for β, *p*-value, and variance inflation factor (VIF). Regression assumptions were assessed using residual Q-Q and residual-versus-fitted plots, the Shapiro–Wilk test of residuals, the Breusch–Pagan test, and inspection of leverage and Cook’s distance. Complementary analyses were performed to verify the robustness of the main model. The Y Balance Test score was added to the main model to examine whether dynamic balance provided additional information about SJFT total beyond countermovement jump and judogi-grip isometric pull-up. Additional models were also tested separately adjusting for sex, body mass, and age. These models were intended as sensitivity analyses rather than as a fully adjusted model; the covariates were entered separately to preserve model parsimony given the sample size. These complementary analyses were used to verify whether the interpretation of the main model remained stable after considering relevant sample characteristics. Intra-session reliability was assessed using the two-way absolute agreement intraclass correlation coefficient based on single measures and Cronbach’s alpha for SJ, CMJ, MBT-SC, and MBT-CM. The significance level was set at α < 0.05. All analyses were performed using Jamovi software, version 2.6.26.

## 3. Results

Table 1 presents the sample characteristics and performance in the physical, functional, and judo-specific tests. The athletes showed mean values of 35.86 ± 7.09 cm in the SJ, 36.96 ± 7.60 cm in the CMJ, 14.97 ± 5.61 repetitions in the DPU, and 59.63 ± 16.67 s in the IPU. In the SJFT, the mean total number of throws was 29.06 ± 2.11 repetitions, with a mean SJFT index of 12.42 ± 1.08. Minimum and maximum values were included to characterize the performance range of the sample.

**Table 1:** Sample characteristics and physical, functional, and SJFT performance (*n* = 36).

Variable	Mean ± SD	95% CI	Minimum	Maximum
Age (years)	18.08 ± 2.18	17.34 to 18.82	15	22
Stature (cm)	171.53 ± 9.71	168.24 to 174.81	150	188
Body mass (kg)	75.04 ± 21.51	67.76 to 82.32	49.80	150.00
YBT score (%)	98.68 ± 7.47	96.15 to 101.21	80.60	113.00
SJ (cm)	35.86 ± 7.09	33.46 to 38.25	24.60	50.50
CMJ (cm)	36.96 ± 7.60	34.39 to 39.53	25.30	56.90
MBT-SC (m)	4.94 ± 0.92	4.63 to 5.25	3.20	7.50
MBT-CM (m)	5.10 ± 1.01	4.76 to 5.44	3.20	8.00
DPU (reps)	14.97 ± 5.61	13.07 to 16.87	4	28
IPU (s)	59.63 ± 16.67	53.99 to 65.27	26.30	106.00
SJFT A (reps)	6.39 ± 0.55	6.20 to 6.57	5	7
SJFT B (reps)	11.89 ± 0.78	11.62 to 12.15	10	14
SJFT C (reps)	10.78 ± 1.05	10.42 to 11.13	9	13
SJFT total (reps)	29.06 ± 2.11	28.34 to 29.77	25	34
HR post-SJFT (bpm)	190.11 ± 10.36	186.61 to 193.62	169	213
HR 1 min post-SJFT (bpm)	169.14 ± 13.19	164.68 to 173.60	122	195
SJFT index	12.42 ± 1.08	12.05 to 12.78	9.97	14.81

Note: Values are presented as mean ± standard deviation, 95% confidence interval, minimum, and maximum. SJ = squat jump; CMJ = countermovement jump; MBT-SC = medicine ball throw without countermovement; MBT-CM = medicine ball throw with countermovement; DPU = judogi-grip dynamic pull-up; IPU = judogi-grip isometric pull-up; YBT = Y Balance Test; SJFT = Special Judo Fitness Test; HR = heart rate.

Table 2 presents the associations between neuromuscular/functional indicators and SJFT performance. SJFT total was positively associated with YBT score, SJ, CMJ, DPU, and IPU. MBT-SC and MBT-CM were not significantly associated with SJFT total. For the SJFT index, the direction of the associations was coherent with the interpretation of the index, as better physical performance tended to be associated with lower index values. After Benjamini–Hochberg adjustment, SJ and CMJ remained associated with the SJFT index, whereas IPU was significant only before adjustment.

Table 3 presents the internal associations among SJFT components. The SJFT index was negatively associated with SJFT total and positively associated with both heart rate variables. These results support the interpretation of the SJFT index as a composite measure, in which lower values reflect a more favorable relationship between throwing performance and post-exercise heart rate response.

Based on the observed associations, parsimonious linear models were tested to explain SJFT performance. First, a model including only CMJ was tested because CMJ represents lower-limb power and showed a large association with SJFT total. Then, IPU was added because it represents judogi-grip isometric pulling strength-endurance, a relevant and complementary capacity to lower-limb power in judo. Model fit is presented in Table 4. The model including CMJ alone showed an adjusted R^2^ of 0.389. When IPU was added, the adjusted R^2^ increased to 0.467. Therefore, the main model composed of CMJ and IPU explained approximately 46.7% of the adjusted variance in SJFT total performance.

Table 5 presents the individual coefficients of the predictors included in the regression models. In the main model for SJFT total, CMJ and IPU remained significant when analyzed simultaneously. SJ was not included together with CMJ because both variables represent closely related lower-limb power constructs. Although YBT score was associated with SJFT total in the bivariate analysis, it was not retained in the main model because it did not show an independent contribution when added to the model including CMJ and IPU. The secondary model for SJFT index was globally significant, but the predictors did not remain individually significant. In M2, each 1 cm increase in CMJ was associated with a mean increase of 0.129 throws in SJFT total, while holding IPU constant. Similarly, each 1 s increase in IPU was associated with a mean increase of 0.044 throws, while holding CMJ constant. Although these unit-level coefficients were small, the complete model accounted for 46.7% of the variance in SJFT total performance after adjustment for model complexity.

Diagnostic tests did not indicate violations of residual normality or homoscedasticity in M1–M3. Cook’s distance was below 1 for all observations in these models; however, one observation showed comparatively high influence in the secondary SJFT-index model (M3; Cook’s D = 0.834). After this observation was excluded, M3 remained statistically significant overall (*p* = 0.025), although the individual coefficient estimates and their statistical significance changed, indicating sensitivity to this observation. As complementary analyses, the main model was tested with the addition of YBT score and with separate adjustments for sex, body mass, and age. Adding YBT score slightly increased the adjusted R^2^ from 0.467 to 0.480, but YBT was not significantly associated with SJFT total after adjustment for CMJ and IPU (*p* = 0.183). In the models adjusted separately for sex, body mass, and age, respectively, CMJ remained significantly associated with SJFT total (unstandardized B = 0.141, 0.130, and 0.125; *p* = 0.005, 0.003, and 0.004), as did IPU (B = 0.045, 0.043, and 0.044; *p* = 0.020, 0.026, and 0.021). The added covariates were not significantly associated with SJFT total: sex (male vs. female: B = −0.377, *p* = 0.610), body mass (B = −0.007, *p* = 0.601), and age (B = 0.066, *p* = 0.596). The adjusted R^2^ rounded to 0.455 in all three models.

## 4. Discussion

The present study examined whether practical neuromuscular and functional field tests were associated with judo-specific fitness in young judoka, with the SJFT total number of throws designated as the primary outcome and the SJFT index as a secondary composite outcome. The principal finding was that lower-limb explosive performance and judogi-grip pulling strength-endurance showed the clearest and most consistent relationships with SJFT total. Squat jump and countermovement jump displayed large positive associations with total throws, both judogi-grip pull-up tests were also positively associated, and countermovement jump together with isometric judogi-grip pull-up provided complementary explanatory information for the primary outcome. The standardized coefficients indicated a stronger independent association of CMJ than IPU with SJFT total, whereas the associations with the SJFT index were weaker and less precise. Dynamic balance was associated with total throws at the bivariate level but provided little additional explanatory value after lower-limb power and grip-specific pulling endurance were considered, whereas neither medicine ball throw condition was clearly associated with SJFT total. Associations with the SJFT index were weaker and less stable, being retained after multiplicity adjustment only for the two vertical jump tests. This study extends previous evidence by concurrently evaluating multiple field-based domains, including dynamic balance, directly comparing SJFT total and SJFT index, and identifying a parsimonious CMJ–IPU combination with potential relevance for applied monitoring.

Lower-limb explosive performance was the strongest field-based domain associated with SJFT throwing output. This result is consistent with a cross-sectional study of 94 judoka aged 11–16 years, in which lower- and upper-limb neuromuscular tests were moderately associated with SJFT outcomes and standing long jump together with handgrip strength explained approximately 30% of SJFT performance variance [12]. It also accords with longitudinal observations in young judoka showing that tournament preparation increased jumping power and improved the SJFT index, with jump height inversely related to the index [25]. Among elite adult judoka, vertical jump performance has likewise shown a strong inverse association with the SJFT index and was associated with a greater probability of achieving at least a regular SJFT classification [13]. These findings are plausible because lower-limb strength and power contribute to the rapid accelerations, changes of direction, body positioning, and explosive projection actions that characterize judo-specific efforts [1]. The similar associations observed for squat jump and countermovement jump suggest that the relevant signal was not restricted to one jump strategy but reflected a broader lower-limb explosive capacity. Countermovement jump was retained in the regression as the more parsimonious representative of this domain, and its independent coefficient should be interpreted as an explanatory association across the observed performance range rather than as evidence that a 1 cm training-induced increase would necessarily produce a fixed improvement in SJFT throws.

Judogi-grip pulling strength-endurance constituted the second major domain associated with SJFT total. Both dynamic and isometric pull-up performance showed large positive correlations with total throws, and isometric pull-up remained independently associated after countermovement jump was included in the model. International-level data from cadet and junior judoka show that SJFT and judogi chin-up performances vary systematically by age and sex, supporting the need to interpret these tests as developmentally and sex-sensitive components of the judo physical profile [14]. Young male judoka also demonstrate greater maximal handgrip strength and grip strength-endurance than untrained peers after adjustment for maturational status, consistent with sport-specific adaptation to repeated gripping demands [26]. The present judogi-grip pull-up tests extend beyond generic handgrip assessment by requiring the athlete to maintain a fabric grip while supporting and pulling body mass, which more closely resembles the sustained gripping and pulling constraints embedded in the SJFT. After lower-limb power was controlled for, the independent contribution of isometric pull-up performance suggests complementarity between explosive lower-limb action and upper-body grip-specific endurance rather than redundancy between these capacities. Nevertheless, more than half of the variance in total throws was not accounted for by the model, indicating that judogi-grip pulling strength-endurance and lower-limb explosive performance capture only part of the technical-tactical, coordinative, metabolic, psychological, and pacing-related factors associated with SJFT performance.

The exploratory findings for dynamic balance and medicine ball throwing require a more qualified interpretation. The Y Balance Test was positively associated with SJFT total, but its addition to countermovement jump and judogi-grip isometric pull-up increased adjusted explained variance only marginally, and it did not retain an independent contribution. Evidence regarding the relationship between balance and SJFT outcomes is inconsistent; in elite judoka, balance performance was not associated with either the SJFT index or its classification [13]. Differences in balance protocol, athlete level, outcome definition, and sample composition may explain why balance emerges as a bivariate correlate in some settings but not as an independent correlate of SJFT performance. Conversely, the absence of an association for either medicine ball throw condition diverges from the study of younger judoka by Kons and colleagues, in which seated medicine ball throw performance was among the neuromuscular variables correlated with SJFT outcomes [12]. The null result, however, should be restricted to the specific medicine ball protocols used here and should not be interpreted as evidence that upper-body power is unimportant to judo performance more broadly. One possible explanation is that the seated medicine ball throw limits trunk and lower-limb involvement and may not fully reproduce the coordinated whole-body actions involved in judo throwing.

The secondary analyses further show that the SJFT index should not be treated as interchangeable with the total number of throws. After false-discovery-rate adjustment, only squat jump and countermovement jump remained associated with the index, while the isometric pull-up association was borderline and the two-predictor model explained substantially less variance than the corresponding model for total throws. This weaker explanatory pattern is consistent with previous regression work indicating that laboratory-derived aerobic and anaerobic fitness variables had only limited capacity to predict SJFT variables, with upper-body relative aerobic power accounting for approximately 15% of the variance in the SJFT index [11]. It is also compatible with evidence that SJFT index classification is associated with vertical jump, sprint, and Wingate power but not consistently with handgrip strength or balance [13]. The SJFT elicits contributions from alactic, glycolytic, and aerobic energy systems, reinforcing that its performance metrics integrate more than a single neuromuscular quality [4]. In the present data, the index was related both to total throws and to post-exercise heart rate, as expected from its mathematical construction. Consequently, a field test may show a direct association with throwing output yet a weaker relationship with the index if it contributes little to immediate heart-rate response or recovery. This distinction supports the a priori choice of total throws as the primary outcome and argues for reporting the index and its components separately rather than interpreting the index as a unitary measure of all dimensions of judo fitness.

The complementary models provide some within-sample support for the stability of the central interpretation, because countermovement jump and judogi-grip isometric pull-up remained associated with SJFT total after separate adjustment for sex, body mass, and age, whereas dynamic balance did not become independently informative. However, maturation, body composition, and training experience have explained meaningful proportions of physical and SJFT performance in youth judoka, and physical performance generally increases across developmental categories [6,27]. The non-significant covariate coefficients in this sample should therefore not be interpreted as evidence that these characteristics are irrelevant. Rather, they indicate that the CMJ–isometric pull-up association was not materially altered by the limited covariate adjustments that the present sample size could support.

Several limitations should be considered. The cross-sectional design identifies contemporaneous associations but cannot establish temporal direction or whether changes in jump or grip pull-up performance would improve SJFT outcomes [28]. Testing occurred approximately one week after the athletes returned to regular training; therefore, differences in early-season training load and recovery, as well as residual fatigue from completing several maximal tests in one session, may have influenced performance, particularly the SJFT. The convenience sample included only 36 athletes from one club, nine of whom were female, and covered a broad range of age and body mass. Pooling sexes and developmental stages without assessing biological maturation limited subgroup analyses and generalizability across sex, age, competitive level, and weight category and may have increased unexplained variability and reduced the precision of the regression estimates. Because the CMJ–IPU model was developed after examining the observed associations, it should be considered exploratory. Although parsimonious, the model was not internally optimism-corrected or externally validated, and its explained variance may be overestimated in a small sample [29]. It should therefore be considered preliminary and not used predictively without further validation. Future studies should use larger multicenter cohorts stratified by sex, age, maturation, weight category, and competitive level; repeat assessments across the season; include technical–tactical and training-load covariates; examine whether within-athlete changes in CMJ and judogi-grip endurance track changes in SJFT performance; and evaluate pre-specified models using bootstrap or cross-validation, independent external validation, and competition-derived outcomes.

From an applied perspective, countermovement jump and isometric judogi-grip pull-up can be considered complementary, low-complexity indicators for monitoring physical capacities associated with SJFT throwing output in developmental judo settings. They should not replace the SJFT, because they do not reproduce its integrated displacement, throwing, pacing, and physiological demands, and the present design does not establish that training changes in either test cause changes in judo-specific fitness. Accordingly, the present study cannot establish a meaningful improvement threshold for CMJ or determine whether longitudinal changes in CMJ and IPU are accompanied by changes in SJFT performance. The Y Balance Test may remain useful as part of a broader functional assessment, but the present findings do not support treating it as an independent proxy for SJFT performance. Likewise, medicine ball throwing may retain value for broader upper-body profiling even though it did not inform SJFT total in this sample. Interpretation should emphasize within-athlete trajectories and use age- and sex-appropriate reference values where available, because SJFT and judogi chin-up performance differ across developmental and sex groups [8,9,14]. Finally, SJFT-related physical test performance should not be used in isolation to infer competitive success, because sport-specific fitness tests have limited evidence for predictive validity and have not shown consistent associations with technical–tactical performance in official judo competition [10].

## 5. Conclusions

In young judoka, lower-limb explosive performance and judogi-grip pulling strength-endurance were the field-based domains most consistently associated with the SJFT total number of throws. Countermovement jump and isometric judogi-grip pull-up provided complementary explanatory information and together accounted for nearly half of the adjusted variance in throwing output, whereas dynamic balance did not add independent explanatory value and medicine ball throw performance was not clearly associated with the primary outcome. Relationships with the SJFT index were weaker, supporting separate interpretation of throwing output and the heart-rate-adjusted composite index. These findings identify two practical monitoring indicators associated with judo-specific fitness in this sample while preserving the SJFT as the more specific assessment and emphasizing the need for longitudinal and externally validated research before predictive or causal applications are made.

## Figures and Tables

**Table 2:** Associations between neuromuscular/functional indicators and SJFT performance (*n* = 36).

Predictor	SJFT Total r (95% CI)	*p*	q BH	SJFT Index r (95% CI)	*p*	q BH
YBT score (%)	0.501 (0.206 to 0.712)	0.002	0.003	−0.316 (−0.584 to 0.014)	0.060	0.106
SJ (cm)	0.670 (0.438 to 0.818)	<0.001	<0.001	−0.438 (−0.670 to −0.128)	0.008	0.035
CMJ (cm)	0.637 (0.390 to 0.799)	<0.001	<0.001	−0.424 (−0.660 to −0.111)	0.010	0.035
MBT-SC (m)	0.277 (−0.057 to 0.555)	0.102	0.119	−0.187 (−0.486 to 0.151)	0.275	0.320
MBT-CM (m)	0.197 (−0.140 to 0.494)	0.249	0.249	−0.139 (−0.447 to 0.199)	0.419	0.419
DPU (reps)	0.514 (0.224 to 0.721)	0.001	0.002	−0.264 (−0.545 to 0.071)	0.120	0.168
IPU (s)	0.579 (0.310 to 0.763)	<0.001	<0.001	−0.381 (−0.630 to −0.060)	0.022	0.051

Note: Values represent Pearson correlation coefficients with 95% confidence intervals. q BH = *p*-value adjusted using the Benjamini–Hochberg procedure within each outcome. YBT = Y Balance Test; SJ = squat jump; CMJ = countermovement jump; MBT-SC = medicine ball throw without countermovement; MBT-CM = medicine ball throw with countermovement; DPU = judogi-grip dynamic pull-up; IPU = judogi-grip isometric pull-up; SJFT = Special Judo Fitness Test. Higher SJFT total and lower SJFT index values indicate better performance in their respective outcomes.

**Table 3:** Internal associations among SJFT components (*n* = 36).

Component 1	Component 2	ρ	*p*
SJFT total	SJFT index	−0.676	<0.001
HR post-SJFT	SJFT index	0.488	0.003
HR 1 min post-SJFT	SJFT index	0.463	0.004
HR post-SJFT	HR 1 min post-SJFT	0.721	<0.001

Note: Values represent Spearman correlation coefficients. ρ = Spearman’s rho; SJFT = Special Judo Fitness Test; HR = heart rate. Lower SJFT index values indicate better judo-specific fitness.

**Table 4:** Model fit for linear regression models of SJFT outcomes (*n* = 36).

Outcome	Model	Predictors Included	R	R^2^	Adjusted R^2^	F	Model *p*
SJFT total	M1	CMJ	0.637	0.406	0.389	23.2	<0.001
SJFT total	M2 main	CMJ + IPU	0.705	0.497	0.467	16.3	<0.001
SJFT index	M3 secondary	CMJ + IPU	0.467	0.218	0.171	4.60	0.017

Note: R^2^ and adjusted R^2^ refer to the complete model. SJFT = Special Judo Fitness Test; CMJ = countermovement jump; IPU = judogi-grip isometric pull-up.

**Table 5:** Regression coefficients for the linear regression models (*n* = 36).

Outcome	Model	Predictor	B	SE	β	95% CI for β	*p*	VIF
SJFT total	M1	CMJ (cm)	0.177	0.037	0.637	0.369 to 0.906	<0.001	1.00
SJFT total	M2 main	CMJ (cm)	0.129	0.040	0.464	0.174 to 0.753	0.003	1.33
SJFT total	M2 main	IPU (s)	0.044	0.018	0.348	0.059 to 0.638	0.020	1.33
SJFT index	M3 secondary	CMJ (cm)	−0.044	0.025	−0.312	−0.673 to 0.050	0.088	1.33
SJFT index	M3 secondary	IPU (s)	−0.015	0.012	−0.225	−0.587 to 0.136	0.213	1.33

Note: B = unstandardized coefficient; SE = standard error; β = standardized coefficient; CI = confidence interval; VIF = variance inflation factor; SJFT = Special Judo Fitness Test; CMJ = countermovement jump; IPU = judogi-grip isometric pull-up. The confidence interval refers to β.

## Data Availability

The data presented in this study are available upon reasonable request from the corresponding author. The data are not publicly available due to privacy and ethical restrictions related to the potential identification of the young judoka included in the study.
